# Prevalence and correlators of burnout among health professionals during different stages of the COVID-19 pandemic in China

**DOI:** 10.3389/fpsyt.2023.1156313

**Published:** 2023-04-26

**Authors:** Zhengshan Qin, Zhehao He, Qinglin Yang, Zeyu Meng, Qiuhui Lei, Jing Wen, Xiuquan Shi, Jun Liu, Zhizhong Wang

**Affiliations:** ^1^Department of Preventive Medicine, School of Public Health at Zunyi Medical University, Zunyi, China; ^2^Department of Epidemiology and Health Statistics, School of Public Health at Guangdong Medical University, Dongguan, China; ^3^Department of Epidemiology and Health Statistics, School of Public Health and Management, Ningxia Medical University, Yinchuan, China; ^4^Department of Epidemiology and Health Statistics, School of Public Health, Zunyi Medical University, Zunyi, Guizhou, China; ^5^The First Dongguan Affiliated Hospital, Guangdong Medical University, Dongguan, China

**Keywords:** mental health, COVID-19, cross-sectional study, health professionals, burnout

## Abstract

**Background:**

Persistently increased workload and stress occurred in health professionals (HPs) during the past 3 years as the COVID-19 pandemic continued. The current study seeks to explore the prevalence of and correlators of HPs' burnout during different stages of the pandemic.

**Methods:**

Three repeated online studies were conducted in different stages of the COVID-19 pandemic: wave 1: after the first peak of the pandemic, wave 2: the early period of the zero-COVID policy, and wave 3: the second peak of the pandemic in China. Two dimensions of burnout, emotional exhaustion (EE) and declined personal accomplishment (DPA), were assessed using Human Services Survey for Medical Personnel (MBI-HSMP), a 9-item Patient Health Questionnaire (PHQ-9), and a 7-item Generalized Anxiety Disorder (GAD-7) to assess mental health conditions. An unconditional logistic regression model was employed to discern the correlators.

**Results:**

There was an overall prevalence of depression (34.9%), anxiety (22.5%), EE (44.6%), and DPA (36.5%) in the participants; the highest prevalence of EE and DPA was discovered in the first wave (47.4% and 36.5%, respectively), then the second wave (44.9% and 34.0%), and the third wave had the lowest prevalence of 42.3% and 32.2%. Depressive symptoms and anxiety were persistently correlated with a higher prevalence risk of both EE and DPA. Workplace violence led to a higher prevalence risk of EE (wave 1: OR = 1.37, 95% CI: 1.16–1.63), and women (wave 1: OR = 1.19, 95% CI: 1.00–1.42; wave 3: OR =1.20, 95% CI:1.01–1.44) and those living in a central area (wave 2: OR = 1.66, 95% CI: 1.20–2.31) or west area (wave 2: OR = 1.54, 95% CI: 1.26–1.87) also had a higher prevalence risk of EE. In contrast, those over 50 years of age (wave 1: OR = 0.61, 95% CI: 0.39–0.96; wave 3: OR = 0.60, 95% CI: 0.38–0.95) and who provided care to patients with COVID-19 (wave 2: OR = 0.73, 95% CI: 0.57–0.92) had a lower risk of EE. Working in the psychiatry section (wave 1: OR = 1.38, 95% CI: 1.01–1.89) and being minorities (wave 2: OR = 1.28, 95% CI: 1.04–1.58) had a higher risk of DPA, while those over 50 years of age had a lower risk of DPA (wave 3: OR = 0.56, 95% CI: 0.36–0.88).

**Conclusion:**

This three-wave cross-sectional study revealed that the prevalence of burnout among health professionals was at a high level persistently during the different stages of the pandemic. The results suggest that functional impairment prevention resources and programs may be inadequate and, as such, continuous monitoring of these variables could provide evidence for developing optimal strategies for saving human resources in the coming post-pandemic era.

## 1. Introduction

Burnout is characterized by emotional and mental exhaustion due to long-term workplace stress and negative job perception and is officially classified as an occupational health syndrome in the 11th revision of the International Classification of Diseases (ICD-11) (1). Conceptionally, it consists of three interrelated dimensions: emotional exhaustion (EE), depersonalization (DP), and declined personal accomplishment (DPA) (2, 3). EE manifests through the loss of enthusiasm for work, feeling helpless, trapped, and defeated; DP is the negative response to other people; DPA refers to inefficiency or the lack of personal achievement (4). Heavy psychological burdens among health professionals (HPs) during outbreaks of SARS-CoV-1, H1N1, MERS-CoV, or Ebola have been reported (5). The prolonged duration of the COVID-19 pandemic has placed unprecedented pressure on HPs who directly participated in procedures including the diagnosis, treatment, and care of patients with COVID-19 (6, 7). It was reported that more than half of HPs had high-stress levels and poor work–family balance during the COVID-19 pandemic (8). Systematic reviews reflected the increase in the prevalence of psychological distress, insomnia, anxiety, depression, and symptoms of post-traumatic stress disorder among health professionals during the current pandemic (7). Several studies have investigated the prevalence of burnout and associated factors among HPs (9–12). A study reported that over one-third of the HPs experienced severe burnout symptoms during the early stage of the pandemic in China (13). It reported age, family income, daily working hours, workload, insufficient protection working in a high-quality hospital, having more years of work experience, having more night shifts and fewer paid vacation days, etc. were associated with burnout among HPs during the pandemic in China (14–16). Although studies observed a positive association between workplace violence and burnout (17, 18), no study reported whether workplace violence affected burnout differently during COVID-19 in China. In brief, most of those studies were conducted at the early stage of the pandemic. It is unclear whether the stressful impact persisted as the pandemic continued.

Although strenuous efforts have been made to control the pandemic worldwide, the situation had no signs of improving until early 2022 (19). In the past 3 years, China adopted lockdown, zero-COVID strategy, and prolonged anti-pandemic measures to fight COVID-19 (20). However, until now, no large studies have been conducted to consistently investigate different phases of the pandemic in burnout among health professionals, as well as modifiable correlators and mitigators of it in mainland China. Moreover, there have been no targeted recommendations put forward for organizations to develop human resource-saving programs and preparedness for future spikes. However, prior research has highlighted emotional exhaustion (EE) as the most sensitive dimension of burnout, with high levels of EE being associated with DP and DPA (20–22). Some studies have suggested that the original three-factor model of the Maslach Burnout Inventory (MBI) can be replaced with one- or two-factor models (23). Hence, we extracted the items of EE and DPA from the MBI in the present investigation to enhance the robustness and feasibility.

Therefore, with the two dimensions of EE and DPA, the current research monitored burnout changes in prevalence and correlators among HPs during the three different stages of the pandemic through a three-wave cross-sectional study.

## 2. Methods

### 2.1. Study design

This study included three repeated online surveys. The first wave of the survey was proceeded 1 month after the first peak of the pandemic in China (27 March and 26 April 2020). The second wave survey was repeated between 27 March and 26 April 2021, when the zero-COVID policy and regular epidemic prevention and control rules were applied nationally. The third wave survey was repeated between 1 April and 30 April 2022, when the second peak of the pandemic happened in China.

### 2.2. Participants and procedure

This online survey was developed following the guidelines of the Checklist for Reporting Results of Internet E-Surveys (12). Individuals who served as physicians, nurses, or medical technicians in any hospitals in mainland China were included. The exclusion criteria were those who were absent from their position for more than 6 months in the past year, cannot access the Internet for any reason, or were unlicensed practitioners. The potentially qualified HPs were invited to join the study through several ways, including social media platforms, such as WeChat, Tencent QQ, and Sina Weibo (tweet in China). Those who responded to the invitation were encouraged to forward the questionnaire link to their colleagues and post it on their own social media networks. Second, an invitation letter was sent to an email list generated by the medical journal association when the email addresses were published with the article.

**Figure 1:**
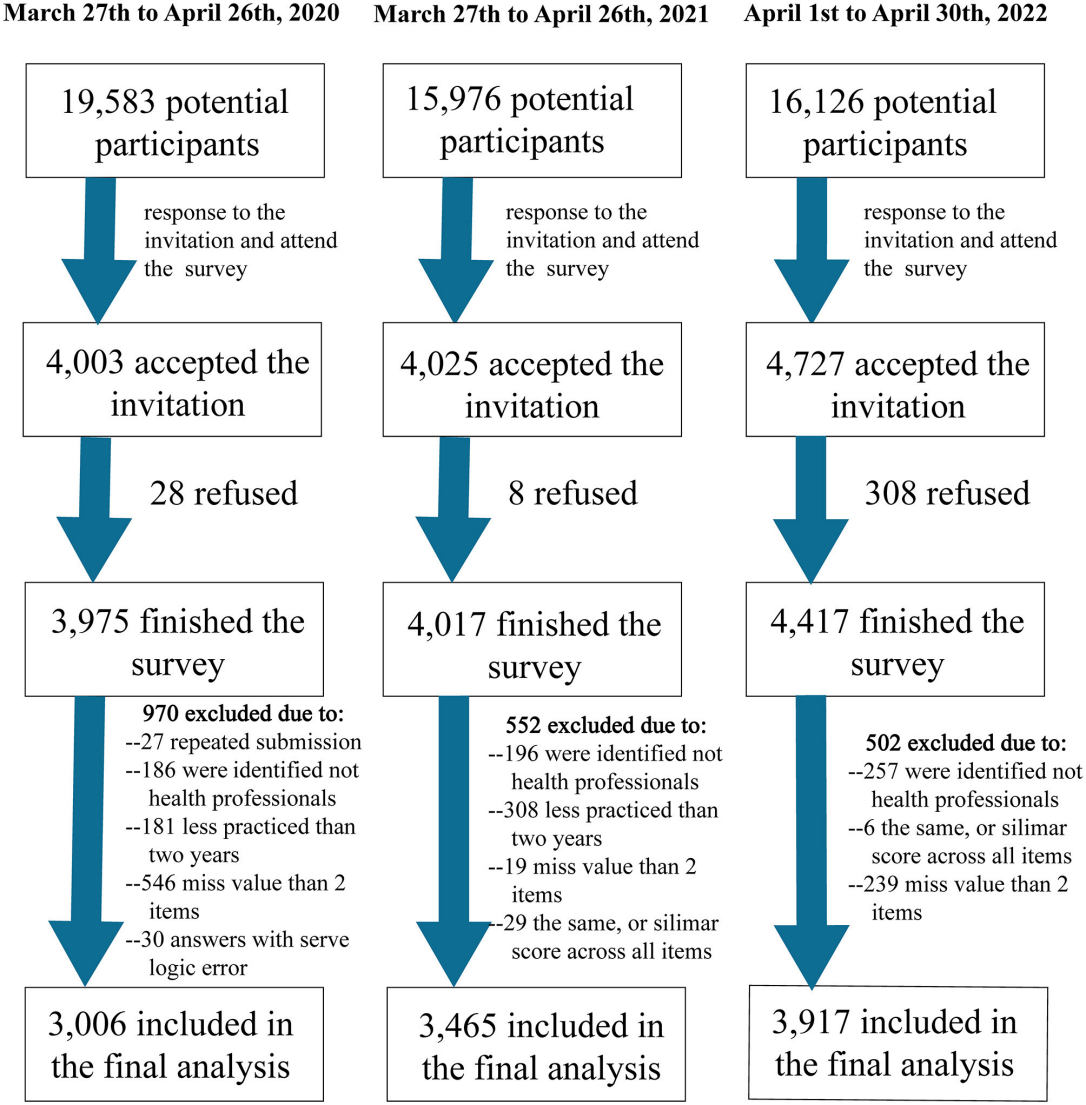
Flow chart of participants' enrollment.

A total of 51,685 potential participants received the invitation to participate (Figure 1). Of them, 12,411 responded and completed the online questionnaire (a response rate of 24.0% in total; 20.2%, 25.1%, and 27.4% in wave 1, wave 2, and wave 3, respectively). Finally, 2,023 participants were excluded during the data cleaning process due to missing values, being identified as non-health professionals, having less than 2 years of practice, and so on, resulting in a sample of 10,388 participants in the analysis.

The survey was conducted on “Wenjuanxin”, an online survey solution provider. The survey link was compatible with multiple devices (including smartphones, laptops, and computers). The survey was anonymous, and online informed consent was acquired by asking participants to tick a box on the device screen. The study was approved by the institutional review board of the Ningxia Medical University (approval #2020-112).

### 2.3. Measurements

Sociodemographic data were collected and included the following variables: age, sex, marital status, educational attainment, ethnicity (Han vs. minorities), ICU/emergency room, physicians/nurses, length of practice, and whether they were direct care providers for patients with COVID-19.

Two dimensions of burnout were assessed using a modified version of the Maslach Burnout Inventory-Human Services Survey for Medical Personnel (MBI-HSMP) (24). As mentioned earlier, we focused on EE and DPA in order to bring more psychometrical robustness and increased feasibility to the present study. Items were scored on a 7-point Likert scale from 0 (never) to 6 (daily), and summed to total scores—higher scores indicate a higher level of burnout. The MBI-HSMP has been shown to have a good validity in HPs previously (25). Cronbach's alpha for this sample was 0.85.

Mental health conditions were assessed by the 9-item Patient Health Questionnaire (PHQ-9) for depressive symptoms and the 7-item Generalized Anxiety Disorder (GAD-7) for anxiety. The Chinese version of the PHQ-9 and GAD-7 scales have excellent psychometrical properties in medical patients (26, 27). Each item on the PHQ-9 and GAD-7 is rated on a 4-point scale indicating the frequency of each symptom in the past 2 weeks, on a scale of 0 (none at all) to 3 (almost daily) (28). We categorized the depressive symptoms as dichotomies depending on the overall score ≥10 and the same with anxiety. Cronbach's alpha in the present sample was 0.91 for PHQ-9 (26) and 0.94 for GAD-7 (27).

Workplace violence (WPV) including the experience of WPV and witnessing WPV was measured using the Chinese version of the Workplace Violence Scale, a scale with proven good reliability and validity to measure violence including physical, mental, and verbal violence that was experienced in the past 12 months (29). The survey provided specific definitions of each type of violence. The individuals who reported any type of violence at least once were defined as violence positive (yes).

### 2.4. Missing values

The mean replacement method was used to replace missing values of sociodemographic variables. We substituted the average of items answered on the scale for the score of missing items when computing scale scores. Those records were deleted when the missing value was more than two items for specific scales and no substitutions were made.

### 2.5. Data analysis

Descriptive statistics were performed by calculating means, standard deviations (SD), and proportions. The chi-square test was employed to test the prevalence of burnout, depression, and anxiety between categorical variables. An unconditional logistic regression model was used to identify the correlators of EE and DPA in different stages of the pandemic. Odds ratios (ORs) with 95% confidence intervals (95% CIs) were calculated under IBM SPSS 23.0. The alpha level was 0.05, with a two-tailed test.

## 3. Results

### 3.1. Epidemiological distribution of the prevalence of burnout

The average age of participants was 35.5 (SD = 8.1) years with a range of 20 to 60 years (In China, a technical secondary nurse can have 2 years of experience at 20 years of age). The average length of practice was 11.0 years (SD = 8.4), ranging from 2 to 40 years. The overall prevalence of depression was 34.9%, and the prevalence of anxiety was 22.5%. As shown in Table 1, the prevalence of EE was 44.6% (4,636/ 10,388), the highest prevalence of EE was found in the first wave (47.4%, 1,425/ 3,006) and then the second wave (44.9%, 1,556/ 3,465), and the third wave had the lowest prevalence of 42.3% (1,655/ 3,917). Those who were aged < 40 years, living in the central areas of China, unmarried, and with a master's degree, and those with a length of practice of < 5 years had a higher prevalence of EE. Similarly, those who work in ICUs or emergency rooms had a higher prevalence of EE (*P* < 0.001), except in wave 1, while those who played a role in psychiatry and nurses had a lower prevalence than other HPs. No statistical significance of the prevalence of EE was found between those directly providing healthcare to patients with COVID-19 and others. Health professionals who reported medical errors, workplace violence, and witnessing workplace violence had a higher prevalence of EE (*P* < 0.001). Furthermore, those with depressive symptoms and anxiety had a much higher prevalence of EE.

**Table 1:** Epidemiological distribution of the prevalence of emotional exhaustion.

	**Wave 1 (*****N*** = **3,006/EE** = **1,425)**				**Wave 2 (*****N*** = **3,465/EE** = **1,556)**				**Wave 3 (*****N*** = **3,917/EE** = **1,655)**				**Total (*****N*** = **10,388/EE** = **4,636)**			
**Variable**	**N**	**%**	χ^2^	**P**	**n**	**%**	χ^2^	**P**	**n**	**%**	χ^2^	**P**	**n**	**%**	χ^2^	**P**
Age group (year)			9.97	0.019			13.47	0.004			19.48	<0.001			37.38	<0.001
≤ 30	526	48.48			490	43.63			579	44.47			1,595	45.44		
30–40	595	47.22			710	47.78			726	43.42			2,031	46.54		
40–50	248	43.36			272	43.66			283	39.25			803	41.91		
≥50	56	39.16			84	36.05			67	30.18			207	34.62		
Sex			0.95	0.330			3.61	0.058			0.06	0.800			3.79	0.051
Male	510	48.62			407	47.71			481	42.57			1,398	46.11		
Female	915	46.76			1,149	43.99			1,174	42.12			3,238	44.02		
Area			23.57	<0.001			39.02	<0.001			3.01	0.222			33.46	<0.001
East	325	45.71			283	35.33			448	43.58			1,056	41.57		
Central	254	58.12			134	49.63			122	45.86			510	52.42		
West	846	45.53			1,139	47.58			1,085	41.36			3,070	44.65		
Marital status			10.01	0.007			2.25	0.324			16.88	<0.001			22.03	<0.001
unmarried	345	52.59			343	46.48			438	48.08			1,126	48.85		
married	1,037	45.76			1,167	44.71			1,144	40.63			3,348	43.53		
div/wid	43	51.19			46	39.32			73	38.42			162	41.43		
Education			42.69	<0.001			13.57	0.001			12.63	0.001			68.50	<0.001
Bachelor	879	43.32			1,221	43.42			1,243	40.86			3,343	42.41		
Master	460	56.58			280	51.76			356	48.30			1,096	52.42		
Ph.D	86	52.44			55	49.11			56	40.58			197	47.58		
Religious affiliation			0.03	0.856			0.01	0.931			2.25	0.133			0.74	0.390
No	1,272	47.46			1,409	44.93			1,504	41.89			4,185	44.49		
Yes	153	46.97			147	44.68			151	46.18			451	45.93		
Length of practice (year)			5.82	0.055			3.73	0.155			7.94	0.019			8.82	0.012
≤ 5	484	49.39			410	46.12			498	44.95			1,392	46.76		
5–10	259	50.00			271	41.56			320	44.08			850	44.83		
≥10	682	45.23			875	45.48			837	40.18			2,394	44.41		
Ethnicity			0.43	0.514			0.03	0.860			3.74	0.053			3.05	0.081
Han	1,225	46.49			1,263	44.83			1,360	43.00			3,878	45.01		
Minorities	170	45.82			293	45.22			295	39.12			758	42.75		
Department			1.36	0.244			20.89	<0.001			3.29	0.070			19.07	<0.001
Other	1,283	47.07			1,358	43.61			1,473	41.78			4,114	43.92		
ICU/emer	142	50.71			198	56.41			182	46.55			522	51.08		
Nurses			15.32	<0.001			5.01	0.025			3.77	0.052			23.94	<0.001
Yes	234	40.14			663	42.80			582	40.25			1,479	41.34		
No	1,191	49.15			893	46.61			1,073	43.42			3,157	46.36		
Psychiatry			2.14	0.144			0.91	0.341			19.78	<0.001			15.39	<0.001
Yes	88	42.51			239	43.06			115	31.34			442	39.15		
No	1,337	47.77			1,317	45.25			1,540	43.38			4,194	46.30		
COVID-19 care			1.59	0.208			4.90	0.027			0.34	0.562			0.06	0.800
Yes	331	49.55			185	40.13			251	43.35			767	44.91		
No	1,094	46.72			1,371	45.64			1,404	42.06			3,869	44.57		
Medical error			19.40	<0.001			23.91	<0.001			36.36	<0.001			86.76	<0.001
Yes	769	51.44			671	50.11			666	48.76			2,106	50.14		
No	656	43.41			885	41.63			989	38.77			2,530	40.89		
WPV			54.21	<0.001			43.12	<0.001			57.97	<0.001			163.42	<0.001
Yes	1,012	52.41			924	50.11			953	48.20			2,889	50.23		
No	413	38.42			632	38.99			702	36.19			1,747	37.69		
Witness WPV			32.47	<0.001			54.38	<0.001			32.94	<0.001			126.82	<0.001
Yes	1,199	50.02			1,119	49.45			1,144	45.65			3,462	48.31		
No	226	37.11			437	36.36			511	36.22			1,174	36.44		
Depression			473.31	<0.001			728.15	<0.001			778.11	<0.001			1,892.80	<0.001
Yes	1,084	65.22			770	82.44			817	79.01			2,671	73.58		
No	341	25.37			786	31.05			838	29.07			1,965	29.08		
Anxiety			479.36	<0.001			577.38	<0.001			626.56	<0.001			1,689.57	<0.001
Yes	678	78.93			604	84.83			630	82.35			1,912	81.85		
No	747	34.79			952	34.58			1,025	32.52			2,724	33.83		

WPV, workplace violence; EE, emotional exhaustion.

Chi-square for the comparison among wave 1, wave 2, and wave 3 is 18.44, P < 0.001.

As shown in Table 2, the overall prevalence of DPA was 34.0% (3,528/ 10,388); the highest prevalence of DPA was found in the first wave (36.5%, 1,098/ 3,006) and then the second wave (34.0%, 1,178/ 3,465), and the third wave had the lowest prevalence of 32.2% (1,252/ 3,917). Similar correlators were found for the prevalence of declined personal accomplishment. In contrast with EE, those with a bachelor's degree and minorities had a higher prevalence of declined personal accomplishment (*P* < 0.05).

**Table 2:** Epidemiological distribution of the prevalence of declined personal accomplishment.

**Variable**	**Wave 1 (*****N*** = **3,006/DPA** = **1,098)**				**Wave 2 (*****N*** = **3,465/DPA** = **1,178)**				**Wave 3 (*****N*** = **3,917/DPA** = **1,252)**				**Total (*****N*** = **10,388/DPA** = **3,528)**			
	**N**	**%**	χ^2^	**P**	**n**	**%**	χ^2^	**P**	**n**	**%**	χ^2^	**P**	**n**	**%**	χ^2^	**P**
Age group (year)			28.33	<0.001			13.03	0.005			42.79	<0.001			76.42	<0.001
≤ 30	451	41.57			407	36.24			475	36.48			1,333	37.98		
30–40	438	34.76			522	35.13			549	32.83			1,509	34.58		
40–50	167	29.20			177	28.41			186	25.80			530	27.66		
≥50	42	29.37			72	30.90			42	18.92			156	26.09		
Sex			0.15	0.701			2.77	0.096			2.33	0.127			0.18	0.673
Male	388	36.99			310	36.34			341	30.18			1,039	34.27		
Female	710	36.28			868	33.23			911	32.69			2,489	33.84		
Area			0.39	0.822			9.26	0.010			4.05	0.132			6.95	0.031
East	263	36.99			239	29.84			307	29.86			809	31.85		
Central	164	37.53			87	32.22			79	29.70			330	33.92		
West	671	36.11			852	35.59			866	33.02			2,389	34.75		
Marital status			10.64	0.005			0.88	0.643			5.73	0.057			12.13	0.002
unmarried	275	41.92			258	34.96			319	35.02			852	36.96		
married	795	35.08			877	33.60			879	31.21			2,551	33.16		
div/wid	28	33.33			43	36.75			54	28.42			125	31.97		
Education			0.48	0.785			1.02	0.601			13.99	0.001			7.59	0.023
Bachelor	749	36.91			967	34.39			1,016	33.40			2,732	34.66		
Master	292	35.92			175	32.35			204	27.68			671	32.09		
Ph.D	57	34.76			36	32.14			32	23.19			125	30.19		
Religious affiliation			0.93	0.335			0.40	0.529			0.65	0.419			0.36	0.548
No	971	36.23			1,061	33.83			1,154	32.14			3,186	33.87		
Yes	127	38.96			117	35.56			98	29.97			342	34.83		
Length of practice (year)			25.87	<0.001			6.70	0.035			21.04	<0.001			49.60	<0.001
≤ 5	406	41.43			332	37.35			395	35.65			1,133	38.06		
5–10	208	40.15			223	34.20			258	35.54			689	36.34		
≥10	484	32.10			623	32.38			599	28.76			1,706	30.93		
Ethnicity			0.40	0.528			10.19	0.001			1.70	0.192			7.54	0.006
Han	957	36.32			923	32.77			996	31.49			2,876	33.38		
Minorities	141	38.01			255	39.35			256	33.95			652	36.77		
Department			0.03	0.868			3.47	0.063			0.84	0.359			2.32	0.128
Other	997	36.57			1,043	33.49			1,119	31.74			3,159	33.73		
ICU/emer	101	36.07			135	38.46			133	34.02			369	36.11		
Nurse			13.29	<0.001			4.80	0.028			26.73	<0.001			31.06	<0.001
Yes	251	43.05			557	35.96			535	37.00			1,343	37.53		
No	847	34.96			621	32.41			717	29.02			2,185	32.09		
Psychiatrist			0.01	0.954			0.83	0.362			0.01	0.935			0.33	0.569
Yes	76	36.71			198	35.68			118	32.15			392	34.72		
No	1,022	36.51			980	33.68			1,134	31.94			3,136	33.87		
COVID-19 care			1.87	0.172			2.76	0.097			1.59	0.207			4.53	0.033
Yes	229	34.28			141	30.59			172	29.71			542	31.73		
No	869	37.19			1,037	34.52			1,080	32.35			2,986	34.40		
Medical error			4.16	0.041			2.34	0.126			0.07	0.795			5.51	0.019
Yes	573	38.33			476	35.55			433	31.70			1,482	35.29		
No	525	34.75			702	33.02			819	32.11			2,046	33.06		
WPV			0.43	0.51			0.32	0.570			5.40	0.02			2.71	0.100
Yes	697	36.10			619	33.57			598	29.94			1,914	33.16		
No	401	37.30			559	34.48			654	33.71			1,614	34.81		
Witness WPV			9.41	0.002			13.27	<0.001			13.25	<0.001			28.76	<0.001
Yes	843	35.17			721	31.86			750	36.48			2,314	34.46		
No	255	41.87			457	38.02			502	35.58			1,214	37.68		
Depression			99.49	<0.001			45.51	<0.001			76.47	<0.001			230.19	<0.001
Yes	738	44.40			401	42.93			443	42.84			1,582	43.58		
No	360	26.79			777	30.70			809	28.06			1,946	28.80		
Anxiety			58.52	<0.001			44.24	<0.001			39.24	<0.001			148.58	<0.001
Yes	405	47.15			317	44.52			317	41.44			1,039	44.48		
No	693	32.28			861	31.27			935	29.66			2,489	30.91		

WPV, workplace violence; DPA, declined personal accomplishment.

Chi-square for the comparison among wave 1, wave 2, and wave 3 is 15.80, P < 0.001.

### 3.2. Multivariate logistic regression

**Figure 2:**
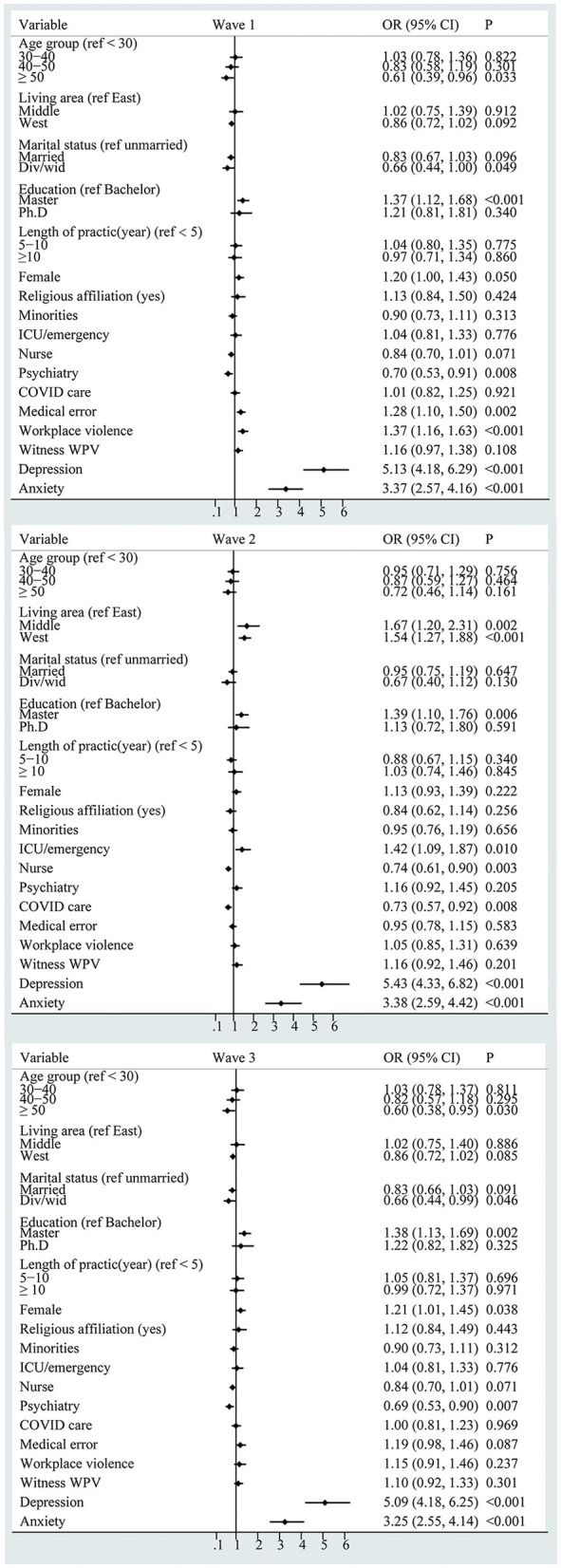
Forest plot of the correlators of emotional exhaustion (WPV: workplace violence).

As shown in Figure 2, slight heterogeneity among the three separate samples was identified in the correlators of burnout. In wave 1 and wave 3 samples, the logistic regression model revealed ages over 50 years had a lower prevalence risk of EE (wave 1: OR = 0.61, 95% CI: 0.39–0.96; wave 3: OR = 0.60, 95% CI: 0.38–0.95). Women had a higher prevalence risk (wave 1: OR = 1.19, 95% CI: 1.00–1.42; wave 3: OR =1.20, 95% CI: 1.01–1.44). In the wave 2 sample, the logistic regression model revealed that living in the central areas (OR = 1.66., 95% CI: 1.20–2.31) and west areas (OR = 1.54, 95% CI: 1.26–1.87) had a higher prevalence risk of EE. Health professionals who directly provide care to patients with COVID-19 had a lower prevalence risk of EE (OR = 0.73, 95% CI: 0.57–0.92). Furthermore, workplace violence led to a higher risk of EE (wave 1: OR = 1.37, 95% CI: 1.16–1.63). Holding a master's degree, depression, and anxiety persistently correlated with a higher risk of EE in all three samples.

**Figure 3:**
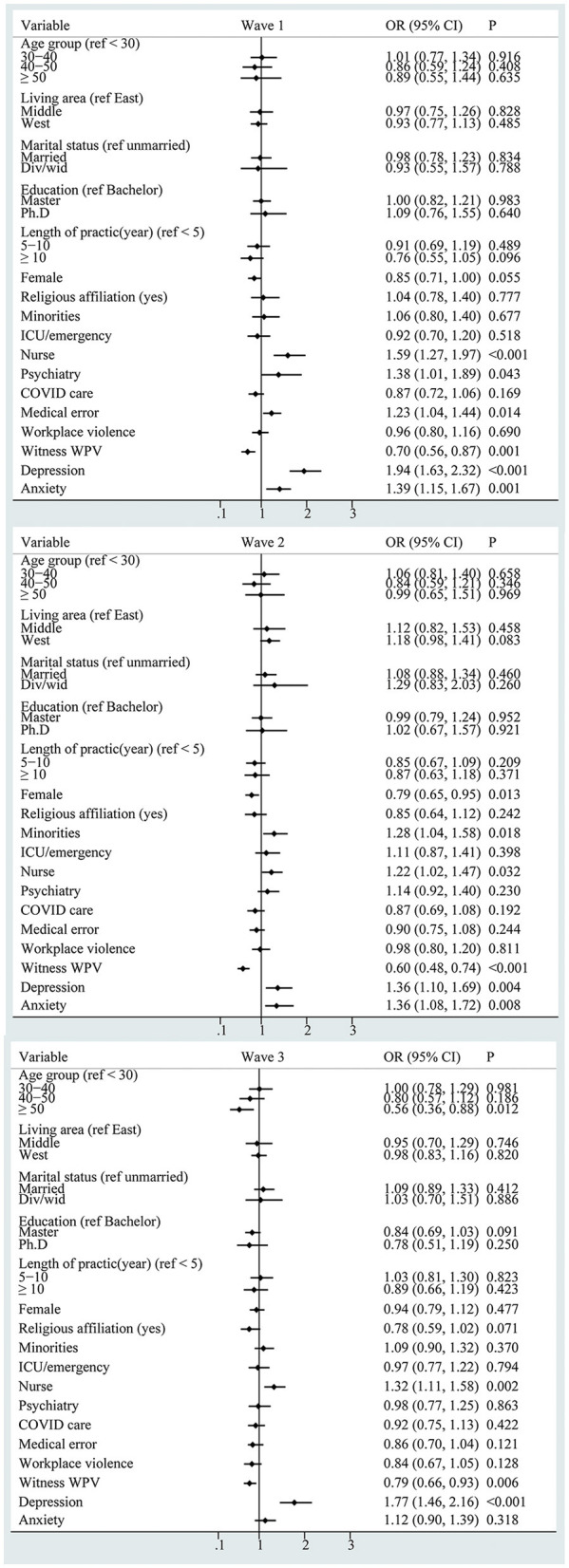
Forest plot of the correlators of declined personal accomplishment (WPV: workplace violence).

The correlators of DPA are shown in Figure 3. Working in the psychiatry section had a higher risk of DPA (OR = 1.38, 95% CI: 1.01–1.89) in wave 1, minorities had a higher risk of DPA (OR = 1.28, 95% CI: 1.04–1.58) in wave 2, and being aged over 50 years had a lower risk of DPA (OR = 0.56, 95% CI: 0.36–0.88) in wave 3. Overall, being a nurse, depression, and anxiety persistently correlated with a higher risk of DPA in all three samples.

## 4. Discussion

During the COVID-19 pandemic, the workload and work stress of health professionals increased dramatically (30). Burnout as a key indicator of functional impairment has been reported repeatedly in the past 3 years (30, 31). To the best of our knowledge, this is one of the first studies that monitored this functional impairment during three stages of COVID-19 among Chinese HPs. This study found high levels of EE and DPA among health professionals during three different stages of the pandemic. There are several possible explanations for the increased risk of burnout. First, the early stage of the COVID-19 pandemic, the lack of resources, and the rapidly increasing cases overloaded health professionals, leading to an increased risk of burnout. Second, 1 year after the pandemic, uncertainty around available resources and the evolution of the virus variants continued to challenge the health system (32). At this stage, strict restrictive measures were adopted in line with the zero-COVID policy (33). Health professionals experienced acute staffing shortages due to the huge efforts on the citywide test-trace-isolate, and following energy-exhausting protocols intended to keep everyone safe (34). In addition, people's daily lives had been disrupted by the long-term control measures against virus spreading, health professionals who had endured emotional and physical exhaustion for more than 2 years, and pandemic fatigue arose at this stage (35).

Adverse mental health outcomes surged during the pandemic (36), leading to functional impairment like burnout. We found that participants with depressive symptoms and anxiety had a higher prevalence of burnout (both EE and DPA) during the different stages of the pandemic. These results are consistent with the findings of other studies. A study conducted in France found a correlation between depression and EE (37). First, burnout and anxiety or depression were mutually influencing, representing that HPs suffering from burnout had a higher level of anxiety or depression, with a remarkable positive correlation between them, and vice versa (1, 38). COVID-19, as a source of stress, inevitably caused anxiety and depression among HPs, leading to their increased risk of EE and DPA, while no association of depression and anxiety was found with DPA in Piedmont's study (39), which in our view might be considered to be influenced by COVID-19 that huge failure in duty by failing to treat patients cause anxiety and depression.

There was an increase in reports of workplace violence attacks against HPs, especially in the early stage of the pandemic. The International Committee of the Red Cross (ICRC) reported 611 incidents of COVID-19-related workplace violence in more than 40 countries during the first 6 months of the pandemic (40). Other studies also found an increase in workplace violence against HPs during the COVID-19 pandemic (41, 42). In the present study, the prevalence of workplace violence was at a high level and was 64.2%, 53.2%, and 50.5% for wave 1, wave 2, and wave 3, respectively. A previous study found that workplace violence against health professionals decreased as the pandemic continued in mainland China (29). Studies have found that workplace violence triggered burnout among HPs (29, 43). Saifur also found workplace violence-exposed nurses were at a greater risk of burnout during the COVID-19 pandemic (44). We also observed a positive association between workplace violence and burnout.

Workplace violence may pose a threat to the life, safety, and dignity of HPs, deteriorating mental health (18). In addition, many studies have also indicated that workplace violence is related to a series of mental health problems, such as depression and anxiety (45, 46), which are relevant to burnout. While no statistical correlation was found in samples of wave 2 and wave 3, not surprisingly, varied correlators were identified in different waves due to the decreased possible exposure in different stages.

However, it was worth noting that the experience of witnessing workplace violence negatively correlated with DPA in all three samples. While several studies conducted among teachers have suggested that witnessing workplace violence is associated with both EE and DPA positively (47–49), differences between teachers and healthcare workers have emerged. Even though experiencing or witnessing workplace violence was prevalent among teachers and healthcare workers (47, 50), workplace violence was mostly perpetrated by students and their parents in the former group (48), whereas in the latter group, most violence was perpetrated by patients or patients' families (50). Furthermore, it should be noted that witnessing workplace violence physically or emotionally, which has not been distinguished in our study, could have a different psychological impact on healthcare workers which indicates that emotional workplace violence could be accepted or normalized by nurses (51). Moreover, a study administered at a medical center found no significant association of ever witnessed workplace violence with burnout (52), indicating that witnessing workplace violence, as an indirect experience where sufferers do not physically get hurt, may have less impact on mental health than experiencing it directly (53).

Those over 50 years old were less likely to suffer from burnout (both EE and DPA), and the reasonable explanation may be senior HPs with extensive experience are more competent in their duties and are likely to receive more respect and adequate rewards while experiencing fewer role conflicts. Furthermore, they are more likely to successfully pace their work, relieve stress, and minimize the risk of job burnout (54). In addition, consistent with many research results (55–59), women showed a higher prevalence risk of EE. Generally, women spent more time on their housework and children than men (60). Moreover, several studies indicated that female HPs were more likely to report having a part-time job (61), and they were more likely to suffer work–family conflict leading to mental problems (62). Additionally, the pressure of HPs increased sharply during the prevalence of COVID-19 (30). Chalhub RÁ (58) and Pappa S (63) reported that female HPs had a higher risk of psychological distress and sleep disruption under stressful situations. This is also true for Chinese female HPs during the current public health crisis (6). The combination of all these factors contributed to a higher level of stress in female HPs. Therefore, more care and support should be given to female HPs.

While, in the wave 2 sample, the logistic regression model revealed that living in central and western areas had a higher prevalence risk of EE, health professionals who directly provide care to patients with COVID-19 had a lower prevalence risk of EE. The pandemic has spread throughout the country since 2019 (62). However, due to the unequal distribution of medical resources, HPs in the central and western regions faced greater difficulties (64, 65). That may be the reason why HPs in central and western regions had a higher risk of EE. Compared with 2020 (the outbreak period of COVID-19) and 2022 (the re-explosion period), HPs involved in COVID-19 work had a higher job satisfaction because of the better control of the pandemic and the use of effective means in 2021. Compared with other professions, nurses were more likely to suffer from DPA (66, 67). For one thing, the shortage of HPs has been a global health system concern in recent years (68). Similar to other nations, China faced the challenge of a nurse resources shortage (17), which inevitably caused an overload of nurses and this problem was significantly magnified during the pandemic. For one thing, the increase in workload made nurses more prone to burnout (69). For another thing, nurses had more direct contact with patients in their daily work. The intensive patient–healthcare worker relationship in China has burdened the nurses with increased workload (68). Furthermore, nurses are overburdened by excessive demands and claim that their work is often stressful, leading to physical and mental exhaustion (70). As a result, some findings call for actions to strengthen communication and organizational support to increase the accomplishment of nurses (71, 72).

### 4.1. Limitations

Several limitations exist in the present study. First, a consequence of the cross-sectional design is that it prevents causal inference; therefore, prospective studies are needed to identify predictors of burnout among health professionals. Second, the convenience sample here requires cautious generalization to service members in the whole nation and other areas outside of China. Third, other potential factors, including the type of hospital, social support, media publicity, and workloads, were not evaluated when exploring the correlators of burnout, which may lead to overestimation or underestimation of the differences between the three stages. Finally, the accuracy of self-reported measures cannot be guaranteed in cases where external factors may influence reporting (even though the survey was anonymous).

## 5. Conclusion

In conclusion, this three-wave cross-sectional study revealed the prevalence of burnout among health professionals at a high level persistently during the different stages of the pandemic. The correlators of burnout varied in dimensions and in stages of the pandemic. These results suggest that current health professionals' functional impairment prevention resources and programs may be inadequate. Considering the high level of uncertainty of the pandemic, continuous monitoring of these variables could provide evidence for developing optimal strategies for saving human resources in the coming post-pandemic era.

## Data availability statement

The original contributions presented in the study are included in the article/supplementary material, further inquiries can be directed to the corresponding authors.

## Ethics statement

The studies involving human participants were reviewed and approved by Institutional Review Committee of Ningxia Medical University. The patients/participants provided their written informed consent to participate in this study.

## Author contributions

ZW and JL: conceptualization and methodology. ZW: data curation and project administration. ZQ and ZH: writing—original draft. JL, XS, and ZW: funding acquisition and writing–reviewing and editing. ZQ, QY, and ZM: formal analysis. JL, JW, and XS: data collection and visualization. All authors have read and agreed to the published version of the manuscript.
